# When Visual Consistency Persuades: Multimodal Congruence, Health Risk, and Health Information Engagement in Digital Media

**DOI:** 10.3390/bs16101841

**Published:** 2026-10-08

**Authors:** Ruiqian Yang, Rong Du, Shanzhuang Zhang

**Affiliations:** School of Economics and Management, Xidian University, Xi’an 710126, China

**Keywords:** image–text congruence, inter-image coherence, topic health risk, digital persuasion, health communication, selective exposure

## Abstract

Visual content is central to online health communication, yet little is known about how multimodal relationships are associated with audiences’ click-through choices. Drawing on processing fluency and risk communication perspectives, this study distinguishes image–text congruence from inter-image coherence and examines their linear, nonlinear, and risk-contingent associations with click-through rate (CTR). We analyze 34,341 health-related articles published by 2184 authors. Clicks are modeled relative to impressions using beta-binomial regression with author and publication-date fixed effects. Image–text congruence is positively associated with the probability that an impression converts into a click, whereas inter-image coherence has an inverted U-shaped association with this probability. Moderate coherence is associated with the highest CTR, while excessive similarity corresponds to declining CTR. Higher topic health risk corresponds to a stronger association between image–text congruence and CTR and a weaker high-coherence decline. The results remain consistent across alternative model specifications, visual measures, outlier exclusion, and different observation windows. By distinguishing cross-modal congruence from within-modal coherence, this study clarifies their different persuasive functions and demonstrates that their associations with click-through choice depend on topic health risk. These findings inform research on digital persuasion and the design of online health content.

## 1. Introduction

Digital media have become an important source of health knowledge, making online health information seeking an increasingly routine practice (Jia et al., 2021; Wang et al., 2021). On attention-driven digital platforms, however, users distribute their attention unevenly across elements of a content preview (Mayer et al., 2024). Whether an impression translates into an actual click depends not only on the article’s topic but also on how the information is presented and how users allocate their limited cognitive resources (Oktay et al., 2021). This selection process is particularly consequential because online health searches can involve escalating health concerns, while cyberchondria is associated with health anxiety (Schenkel et al., 2021; White & Horvitz, 2009). Identifying the interface cues that prompt users to move from an impression to an actual click is therefore not merely a question of user engagement on digital platforms; it also concerns a critical gateway to health communication.

Before users decide whether to open an article, its full text remains unavailable. At this stage, the article preview, composed of a headline and accompanying images, offers visible cues that can influence the decision to open the article (Kuiken et al., 2017; Zillmann et al., 2001). Headlines summarize the central topic, foreground the relevant health issue, and signal the potential informational value of the article, thereby shaping content selection (Kuiken et al., 2017). Images can influence selective reading and click-through intentions in related contexts (Y. Yang et al., 2025; Zillmann et al., 2001). Prior research further suggests that image content and its alignment with the topic of a post can affect user engagement in particular platform contexts (Y. Li & Xie, 2020).

When an article displays multiple images, users encounter a visual configuration created by the images as a set. People can extract summary characteristics from multiple objects and form an ensemble representation of them (Alvarez, 2011; Whitney & Yamanashi Leib, 2018). The effect of a multi-image preview therefore may depend on more than the sum of the attributes of its individual images. In social-media posts, image richness has been associated with different forms of user engagement (Zhao et al., 2023). However, repeated images may add little new information, whereas visual variety can affect set processing and, in large assortments, contribute to choice complexity (Chowdhury et al., 2011; Townsend & Kahn, 2014). Research on multi-image previews must therefore consider not only what each image depicts but also how the headline and images, as well as the images themselves, form an integrated information structure.

Research on digital content has examined headline features and individual image characteristics (Kuiken et al., 2017; Y. Li & Xie, 2020). Those studies leave open how a headline and several cover images operate together within one article preview. Consistency is inherently relational: it does not reside in a headline or an image alone but instead reflects the extent to which different informational cues support one another around a shared theme. Processing fluency theory suggests that stimuli which are easier to recognize, organize, and understand generally elicit more favorable evaluations (Reber et al., 2004). Consistent with this view, previous research has shown that coordination between images and text can facilitate information processing and improve communication outcomes (Van Rompay et al., 2010). When images accompanying a review correspond to its textual content, they can also help users verify the object described in the text, thereby increasing the review’s perceived helpfulness (Ceylan et al., 2024).

Treating all forms of consistency as manifestations of the same processing-fluency mechanism, however, obscures both the informational elements between which consistency occurs and the function it serves in users’ pre-click evaluations (Heckler & Childers, 1992; Liu et al., 2025). We therefore distinguish between two forms of cover consistency. The first is image–text congruence, defined as the average degree of semantic correspondence between the article’s headline and its multiple cover images. The headline and images can convey related cues about the article’s topic (Ceylan et al., 2024; Van Rompay et al., 2010). Image–text congruence thus is conceptualized here as serving a thematic verification function, which may help users assess the article’s topic before clicking. The second is inter-image coherence, defined as the degree of similarity among the cover images in their semantic content and visual representations. Inter-image coherence is conceptualized here as serving a visual-configuration function by describing how strongly the images form a recognizable set around a shared theme.

Prior research indicates that different forms of correspondence between text and images may produce distinct patterns of user engagement (Liu et al., 2025). The relationships between user responses and either image–text relevance or emotional congruence may also be nonlinear (J. Cao et al., 2025; H. Li et al., 2026). These studies, however, address cross-modal relationships between text and images, rather than directly examining how multiple images are organized within an article preview.

The effects of these two forms of cover consistency may also depend on the level of health risk associated with an article’s topic. Risk judgments shape individuals’ information needs, willingness to seek information, and modes of information processing and may motivate both information seeking and information avoidance (Z. J. Yang et al., 2014; Zhou et al., 2023). The way health information is presented can also affect how users understand a risk and its implications for action (Woloshin et al., 2023). Moreover, health anxiety, the stage of the information-seeking process, and arousal can alter the allocation of attention (Ke et al., 2023; Mather & Sutherland, 2011). We therefore treat topic health risk as a contextual variable that may condition the effects of both forms of cover consistency on click-through choice, allowing us to examine how health risk changes users’ reliance on specific multimodal relationships.

The preceding discussion suggests that headlines and images are not independent components of an article preview. Instead, the relationships among them may jointly shape users’ selection of health information. Accordingly, this study asks how the consistency of multimodal article previews influences users’ decisions to open health information articles and how topic health risk shapes this process. We address this overarching question through three specific research questions:

**RQ1:** How does image–text congruence influence users’ decisions to open health information articles?

**RQ2:** How does inter-image coherence influence users’ decisions to open health information articles?

**RQ3:** How does topic health risk moderate the relationships between the two forms of cover consistency and users’ click-through choices?

To answer these questions, we examine health articles displayed with multiple cover images and track their impressions and clicks for nearly a month. After applying the sample-selection criteria, the final analytical sample comprises 34,341 articles published by 2184 authors. We measure image–text congruence using Chinese-CLIP to calculate the average semantic similarity between an article’s headline and its three cover images. We measure inter-image coherence as the average pairwise similarity among the three images. The empirical analysis treats article clicks as the outcome of user choice conditional on opportunities for exposure. We estimate beta-binomial models with author and publication-date fixed effects while controlling for image attractiveness, headline characteristics, health source cues, and other relevant factors.

The results reveal distinct patterns of association between the two forms of cover consistency and users’ click-through choices. First, image–text congruence is positively associated with click-through probability, and this positive association becomes stronger as topic health risk increases. Second, inter-image coherence exhibits an inverted U-shaped association with click-through probability: the propensity to click initially increases with coherence but subsequently declines. The shape of this relationship also varies with topic health risk. Compared with low-risk topics, high-risk topics exhibit a weaker decline in click-through probability at high levels of inter-image coherence.

This study makes three primary contributions. First, it extends research on digital health information to the selection stage that precedes exposure to an article’s full content. By conceptualizing the multi-image article preview as a compressed interface-level representation of the underlying content and distinguishing platform-provided impressions from user-initiated clicks, the study advances our understanding of engagement processes on digital health platforms. Second, it refines the conceptual structure of multimodal consistency by distinguishing image–text congruence from inter-image coherence. The former primarily serves a thematic verification function, whereas the latter primarily serves a visual-configuration function. This distinction moves consistency research beyond a general notion of correspondence by specifying how different informational relationships shape user choice. Third, the study examines the distinct functional forms and risk-contingent boundaries of these two relationships. By analyzing the linear association of image–text congruence, the potentially nonlinear association of inter-image coherence, and the moderating role of topic health risk, it reveals how the relationship between multimodal message design and click-through choice varies across health-risk contexts.

## 2. Literature Review

### 2.1. Cover Cues in Digital Content Previews

On digital media platforms, users typically encounter a content preview composed of a headline, images, source information, and other interface elements before accessing the full content. Among these elements, headlines and images are particularly important to attract visual attention (J. Cao et al., 2025; Mayer et al., 2024). As a primary verbal cue in an article preview, the headline can influence content selection through its length, wording, syntactic structure, and referential style (Kuiken et al., 2017). Headlines can also create information gaps by withholding key details, thereby generating an incentive to click (Blom & Hansen, 2015). Moreover, negative words in headlines can increase news-consumption rates (Robertson et al., 2023). These findings suggest that a headline does more than indicate what an article is about; it also influences users’ willingness to click by foregrounding a problem and promising potentially valuable information.

As important visual cues in an article preview, images can rapidly convey object- and scene-level information (Fei-Fei et al., 2007). Early communication research demonstrated that news photographs affect readers’ selective exposure to news reports (Zillmann et al., 2001). Images can also shape the selection of and engagement with digital content by conveying information and capturing attention (Koh & Cui, 2022). In crowdfunding, cover images depicting restaurant properties have been shown to increase potential backers’ click-through intentions (Y. Yang et al., 2025). Social media research indicates that the presence of an image, the depiction of people, and the image’s color and content characteristics are associated with user engagement, although these effects vary across platforms (Y. Li & Xie, 2020). Image richness may also have distinct effects on affective, behavioral, and cognitive engagement (Zhao et al., 2023).

Research has increasingly moved beyond examining individual headlines or images to investigating how verbal and visual content are jointly configured. Studies using computer vision and natural language processing have found that the balance between text and images, their semantic relationship, and the number of objects depicted collectively shape social media engagement (Dang et al., 2026). The effectiveness of an article preview therefore depends not only on what the headline and images contain separately but also on how these cues operate together. Research must consequently move beyond isolated cover cues to examine the relational properties among them.

### 2.2. Consistency in Information Presentation

Consistency is not an intrinsic attribute of an individual information element; rather, it is a relational property between two or more cues (Heckler & Childers, 1992; Van Rompay et al., 2010). Its meaning depends on both the elements being compared and the dimension along which they are compared. Semantic relevance, expectancy, visual similarity, and internal coherence should therefore not be treated as interchangeable forms of consistency. Classic advertising research distinguishes between two dimensions of information incongruence: relevancy and expectancy. Relevancy refers to whether a cue contributes to identifying the central theme, whereas expectancy refers to whether the manner in which that cue is presented conforms to an individual’s existing schemas and knowledge structures (Heckler & Childers, 1992). Research on social media has further shown that different combinations of image–text relevancy and expectancy generate distinct effects on commenting and sharing, demonstrating that consistency is not a unidimensional stimulus property (Liu et al., 2025).

Online marketing research has found that when images and text convey congruent symbolic meanings, users can process product information more readily and form more favorable attitudes (Van Rompay et al., 2010). Research on online reviews demonstrates that images can provide visible evidence for textual descriptions. Greater similarity between review photographs and written content facilitates review processing and increases perceived helpfulness (Ceylan et al., 2024). Research on online medical question-and-answer platforms likewise shows that answers are perceived as more helpful when their content matches the linguistic characteristics of the question (Peng et al., 2020). These findings suggest that the value of cross-cue congruence arises not merely from surface-level similarity but also from the extent to which different information elements jointly support users’ judgments. Different forms of congruence may also operate through distinct communication pathways. In social media messages intended to debunk misinformation during public health crises, topic congruence and reputation congruence have different implications for the persuasiveness of cognitive and affective appeals (Zhang et al., 2024). Research on online reviews similarly finds that image accuracy and relevance generally increase review helpfulness (Y. Yang et al., 2023). Overall, cross-cue congruence can shape information processing, diagnostic judgments, and persuasive outcomes, but its effects depend on the specific relational dimension and communication task involved.

In addition to cross-cue relationships between text and images, consistency can arise within a set of elements belonging to the same modality. Research on ensemble perception shows that the human visual system can encode multiple objects as a set and rapidly extract their average characteristics, range, and overall tendency (Alvarez, 2011). Some visual commonality can support coherence, but excessive similarity may create informational redundancy. The marginal communication benefit of additional images declines when they repeat information already conveyed by existing images (Chowdhury et al., 2011). Research on visual complexity consequently distinguishes organized design complexity from disorganized feature complexity. The former can enhance attention and evaluation, whereas the latter is more likely to impair comprehension and attitudes (Pieters et al., 2010). Research on visual perspective across images also shows that multiple perspectives can provide more comprehensive information but may also increase the difficulty of shifting between and integrating scenes; their effects therefore depend on the user’s task goal (Y. Jiang et al., 2014). Information systems research similarly demonstrates that the effects of perceived complexity on user responses vary between goal-directed search and experiential browsing (Nadkarni & Gupta, 2007). Visual order and complexity may also jointly influence pleasure, arousal, and approach behavior (Deng & Poole, 2010). Taken together, these findings indicate that the effects of a multi-element configuration cannot be fully explained by the number of elements or by their individual attributes. They also depend on whether the elements form an integrated whole and how commonality and differentiation are balanced within that configuration.

Traditional accounts based on processing fluency generally predict that consistency produces favorable outcomes (Reber et al., 2004). Other research, however, suggests that consistency and differentiation can each provide distinct forms of value. Research on image–text congruence shows that high congruence facilitates rapid recognition and fluent processing, whereas low congruence may stimulate interest through surprise and elaborative processing, thereby producing a nonlinear effect (J. Cao et al., 2025). Research on novelty and complexity similarly indicates that moderate novelty or cognitive challenge can encourage information processing, whereas stimuli that are overly simple or excessively complex may elicit boredom or confusion (Berlyne, 1970; Graf & Landwehr, 2015). Nonlinear effects of consistency have also been observed on digital platforms. For example, emotional consistency between a cover image and narrative text may exhibit an inverted U-shaped relationship with crowdfunding performance, suggesting that increasing consistency can facilitate processing but may also introduce informational repetition (H. Li et al., 2026). The effects of consistency are also contingent on the information task, content type, and risk context. Disease acuity changes the value of content–context congruence (Peng et al., 2020), while health anxiety, search stage, and task type shape attentional biases during online health information processing (Ke et al., 2023). In summary, the behavioral consequences of consistency cannot be reduced to a simple linear effect. Instead, they may be both nonlinear and substantially dependent on the context in which information is encountered and processed.

## 3. Hypotheses and Research Model

**Figure 1:**
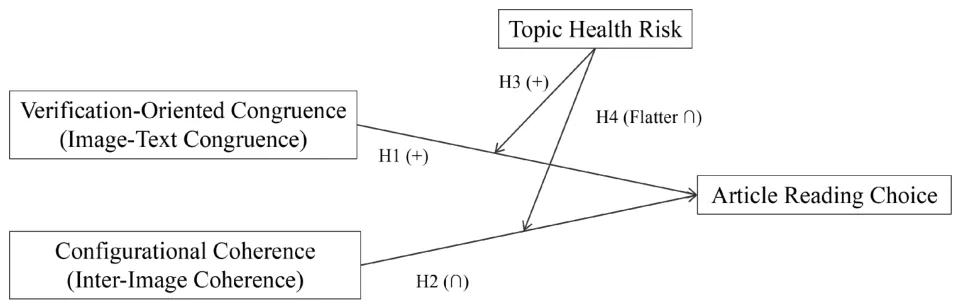
Conceptual model.

Figure 1 presents the conceptual model, which specifies the distinct effects of image–text congruence and inter-image coherence on click-through rate (CTR), as well as the moderating role of topic health risk.

### 3.1. The Main Effect of Verification-Oriented Congruence

Before clicking on an online health article, users must infer from its headline and cover images whether the content is relevant to their current health needs. When the headline and images are semantically congruent, verbal and visual cues reinforce one another, potentially allowing users to identify the article’s topic more readily. Verbal and pictorial information can generate interconnected representations during processing. Image–text congruence facilitates this relational processing and reduces the difficulty of integrating verbal and visual information (Houston et al., 1987; Van Rompay et al., 2010). When an image corresponds to the accompanying text, it also provides visible evidence of the object or experience being described, thereby increasing processing ease and the diagnostic value of the information (Ceylan et al., 2024). Both image content and its correspondence with verbal content can influence user engagement, indicating that image–text relationships offer explanatory value beyond the isolated properties of either cue (Dang et al., 2026; Y. Li & Xie, 2020). Research on online medical question-and-answer platforms similarly shows that alignment between message characteristics and the context of a health problem can increase the perceived value of the information (Peng et al., 2020). Accordingly, greater verification-oriented congruence may make it easier for users to confirm what an article addresses and potentially reduce uncertainty during pre-click evaluation.

Accordingly, we propose:

**Hypothesis** **1** (H1)**.** *Image–text congruence between an article’s headline and its cover images is positively associated with the CTR.*

### 3.2. The Nonlinear Effect of Configurational Coherence

Configurational coherence captures the extent to which multiple images form an organized visual ensemble centered on a common theme. When configurational coherence is low, the images provide little thematic commonality, making it difficult for users to form a stable inference about the content. The human visual system can encode multiple objects as an ensemble and rapidly extract their summary properties, but such processing requires at least some degree of perceptual or conceptual groupability (Alvarez, 2011; Whitney & Yamanashi Leib, 2018). Unstructured visual variation increases integration difficulty, whereas complexity organized around a discernible structure can enhance attention and evaluation (Pieters et al., 2010). Thus, as configurational coherence increases from a low level, the images should become easier to organize into a unified representation, thereby improving the thematic clarity of the cover.

A moderate level of configurational coherence can preserve both thematic unity and informational differentiation. Multiple images may depict different objects, settings, or behaviors associated with the same health issue, enabling users to recognize a shared theme while anticipating that the article will address several complementary aspects of that issue. Research on visual perspective suggests that the effects of presenting information from different perspectives depend on whether those perspectives support the viewer’s processing goal (Y. Jiang et al., 2014). When differences among images serve a common informational purpose, they can therefore provide complementary information without undermining the coherence of the overall visual ensemble.

When configurational coherence becomes excessively high, however, the images may become repetitive, reducing the marginal informational value of each additional image. When additional images merely repeat an emotion, attribute, or benefit already conveyed by other images, their incremental communication value progressively diminishes (Chowdhury et al., 2011). Visual richness and complexity can attract attention and encourage exploration, suggesting that covers offering little informational variation may have limited exploratory value (Z. Sun & Firestone, 2021; Zhao et al., 2023). Theories of novelty and complexity suggest that stimuli require sufficient variation to evoke interest while remaining comprehensible: insufficient differentiation can produce boredom, whereas excessive differentiation can produce confusion (Berlyne, 1970; Silvia, 2005). A moderate level of configurational coherence should therefore balance processing fluency with exploratory value, producing more favorable responses than either very low or very high coherence (Graf & Landwehr, 2015).

In summary, low configurational coherence increases integration difficulty, moderate coherence balances holistic organization with complementary information, and excessive coherence can create informational redundancy and reduce exploratory value.

Accordingly, we propose:

**Hypothesis** **2** (H2)**.** *Inter-image coherence among an article’s cover images has an inverted U-shaped association with the CTR, such that CTR is higher at moderate levels of inter-image coherence than at either low or high levels.*

### 3.3. The Moderating Role of Topic Health Risk in the Effect of Verification-Oriented Congruence

Topic health risk captures both the severity of the potential consequences associated with a health issue and the susceptibility of the general population to that issue. As the potential consequences of a health problem become more severe, users have a greater need to understand the issue adequately. The discrepancy between what they currently know and what they believe they need to know consequently becomes more salient, generating stronger information needs (Griffin et al., 2013; Z. J. Yang et al., 2014). Consistent with this reasoning, perceived risk and information insufficiency have been identified as important drivers of health-risk information seeking (ter Huurne & Gutteling, 2008; Zou et al., 2024).

Higher health risk may affect not only whether users seek information but also how they select it. When confronting a high-risk topic, choosing irrelevant content may delay or impede understanding of the problem. Users therefore have a stronger incentive to identify an article’s topic and potential informational value accurately. Processing goals and risk-related arousal can direct limited attentional resources toward goal-relevant and diagnostically useful cues (Ke et al., 2023; Mather & Sutherland, 2011; Pieters & Wedel, 2007). During pre-click evaluation, image–text congruence may reduce integration difficulty and may help users determine whether an article addresses the health issue relevant to them (Ceylan et al., 2024; Van Rompay et al., 2010). The value of content–context congruence also varies with disease acuteness, further suggesting that health risk can increase the diagnostic value of congruence cues (Peng et al., 2020). Therefore, as topic health risk increases, users should rely more heavily on mutual verification between the headline and cover images, strengthening the positive association between image–text congruence and CTR.

Accordingly, we propose:

**Hypothesis** **3** (H3)**.** *Topic health risk positively moderates the association between image–text congruence and CTR.*

### 3.4. The Moderating Role of Topic Health Risk in the Effect of Configurational Coherence

The association between configurational coherence and CTR depends on the balance between holistic organization and informational differentiation. Topic health risk may alter the relative importance users assign to these two forms of value. As topic health risk increases, users’ informational goals are more likely to center on identifying the problem, understanding the risk, and obtaining relevant information. Research on task orientation shows that goal-directed search places greater emphasis on informational order and processing efficiency than experiential browsing (Nadkarni & Gupta, 2007). Different processing goals also change how users allocate attention across text, images, and other visual elements (Pieters & Wedel, 2007).

Heightened arousal strengthens the attentional advantage of high-priority information while suppressing information assigned lower priority (Mather & Sutherland, 2011). Emotional states characterized by high motivational intensity can also narrow attentional scope, causing individuals to focus more strongly on goal-relevant stimuli (Gable & Harmon-Jones, 2010). Research on attention under threat further shows that threat can reduce distraction by otherwise salient but task-irrelevant reward cues (Kim & Anderson, 2020). Together, these findings suggest that high-risk contexts may increase the value users place on thematic concentration and ease of visual integration. Consequently, although greater configurational coherence reduces differentiation among images, the cost of this redundancy may become less consequential for high-risk topics. As long as the images remain centered on the focal health issue, similar visual information can reinforce thematic concentration and make the cover easier to comprehend and integrate. Users may therefore tolerate a certain degree of visual repetition in exchange for a clearer representation of the health problem. This does not mean that high health risk eliminates the value of visual diversity. When different images provide complementary information relevant to the same health problem, their differences may still facilitate processing, particularly when they support the user’s informational goal (Y. Jiang et al., 2014). Health risk should primarily reduce the value of variation that offers little additional diagnostic information and serves only to create visual novelty. Research on digital platforms likewise suggests that risk can alter the strength of nonlinear congruence effects, thereby changing the relative weight users assign to the benefits and costs of consistency (H. Li et al., 2026). For low-risk topics, high configurational coherence is more likely to reduce CTR because of informational repetition and limited exploratory value. For high-risk topics, users may place greater value on thematic concentration and ease of integration; therefore, the decline in CTR associated with high inter-image coherence should be less pronounced.

Accordingly, we propose:

**Hypothesis** **4** (H4)**.** *Topic health risk moderates the inverted U-shaped association between inter-image coherence and CTR, such that the decline in CTR at high levels of inter-image coherence is weaker for high-risk topics than for low-risk topics.*

## 4. Empirical Study

### 4.1. Data Source and Sample Construction

The data were collected from Toutiao^1^, a major Chinese digital content platform. The initial article pool comprised 46,184 articles, each of which was monitored daily for nearly one month following its publication. We first excluded articles with fewer than three cover images and those with incomplete monitoring records. This screening procedure yielded 36,465 articles published by 4289 authors, corresponding to approximately 1.1 million article-day observations. To ensure that the author fixed effects could be identified from variation across articles published by the same author, we further excluded authors who had published only one article, along with their corresponding 2105 articles. This procedure reduced the sample to 34,360 articles. We then assessed the validity of the outcome variables by requiring that $0\leq {\mathrm{Click}}_{i}\leq {\mathrm{Imp}}_{i}$ and ${\mathrm{Imp}}_{i}>0$, where ${\mathrm{Click}}_{i}$ and ${\mathrm{Imp}}_{i}$ denote the cumulative numbers of clicks and impressions for article *i*, respectively. The final analytical sample consisted of 34,341 articles published by 2184 authors.

Prior research suggests that collective attention to online content typically emerges rapidly after publication and subsequently decays, producing a discernible attention lifecycle (Wu & Huberman, 2007). The monitoring data showed that, after Day 21, the daily growth rates of both clicks and impressions had fallen below 0.5%, indicating that article traffic had entered a plateau phase. By Day 7, cumulative clicks and impressions had reached, on average, 89.82% and 86.29% of their respective Day 21 levels. Day 7 therefore represents a short-term accumulation phase in which most article traffic has already materialized but some subsequent growth remains. The corresponding proportions on Day 1 were 70.40% for clicks and 58.67% for impressions, representing the initial burst phase immediately following publication. We therefore selected Days 1, 7, and 21 as observation points representing distinct stages of the article attention lifecycle. Day 1 outcomes were used in the main analysis, whereas Day 7 and Day 21 outcomes were examined in robustness tests. This design allowed us to assess whether the findings remained consistent across different stages of traffic accumulation.

### 4.2. Key Variables and Measurement

The outcome variable is the cumulative number of article-opening clicks (Click), with the number of impressions (Imp) representing the total opportunities for an article to be clicked. Specifically, we treat the number of clicks as an outcome conditional on the number of impressions and examine the click-through rate (CTR), defined as the probability that users choose to click to open an article after it is displayed to them. The key explanatory variables are image–text congruence (ITC) and inter-image coherence (IIC). ITC captures the degree of cross-modal semantic alignment between an article’s headline and its three cover images, whereas IIC captures the internal coherence of the semantic representations of the three cover images associated with the same article. Topic health risk (THR) serves as the moderating variable and is used to examine whether health risk changes the relationships between the two forms of cover consistency and users’ article click-through choices.

#### 4.2.1. Independent Variables: Chinese-CLIP-Based Congruence Measures

Contrastive Language–Image Pretraining (CLIP) uses contrastive learning to map text and images into a shared semantic embedding space, such that matched image–text representations are positioned closer together, whereas unmatched representations are positioned farther apart (Radford et al., 2021). Building on the CLIP architecture, Chinese-CLIP is pretrained on large-scale Chinese image–text data and is therefore better suited to identifying cross-modal semantic relationships between Chinese headlines and images (A. Yang et al., 2022).

For article *i*, let its headline $T_{i}$ denote the textual input and let $I_{i1}$, $I_{i2}$, and $I_{i3}$ denote its three cover images. Let $f_{T}\left(\cdot \right)$ and $f_{I}\left(\cdot \right)$ represent the text and image encoders of Chinese-CLIP, respectively. We used these encoders to obtain embeddings for the headline and each cover image, applied $L_{2}$ normalization, and then used cosine similarity to quantify semantic correspondence between the resulting representations. At the article level, image–text congruence was defined as the mean cosine similarity between the headline and the three cover images:

(1) $${\mathrm{ITC}}_{i}=\frac{1}{3}\sum_{j=1}^{3}\cos\;\left(f_{T}\left(T_{i}\right),f_{I}\left(I_{ij}\right)\right).$$

Inter-image coherence was measured in the same embedding space by calculating cosine similarity for each of the three unique pairs of cover images. Article-level inter-image coherence was then defined as the mean of these three pairwise similarities:

(2) $${\mathrm{IIC}}_{i}=\frac{1}{3}\sum_{j<k}\cos\;\left(f_{I}\left(I_{ij}\right),f_{I}\left(I_{ik}\right)\right),\;j,k\in \left\{1,2,3\right\}.$$

A higher value of ${\mathrm{ITC}}_{i}$ indicates stronger semantic alignment between the article headline and its three cover images, whereas a lower value indicates greater semantic divergence between the headline and the accompanying visual content. A higher value of ${\mathrm{IIC}}_{i}$ indicates that the three cover images are more concentrated around a common topic and convey more similar semantic content, whereas a lower value indicates greater semantic differentiation among the images.

#### 4.2.2. Moderating Variable: Topic Health Risk

Health risk encompasses both the severity of potential health consequences and the likelihood that individuals may be affected. Health communication research distinguishes severity from susceptibility because they capture different dimensions of risk perception (El-Toukhy, 2015). Consistent with protection motivation theory, threat appraisal is jointly determined by perceived severity and perceived susceptibility or vulnerability (Lahiri et al., 2021). Risk characteristics conveyed by online health information may, in turn, shape users’ information processing and behavioral responses (H. Li et al., 2025).

Accordingly, we define topic health risk (THR) as the combined level of risk associated with the health issue represented in an article’s headline. It comprises two topic-level dimensions. Topic health severity captures the seriousness of the potential adverse consequences of the disease, symptom, health condition, or health behavior mentioned in the headline. Topic health susceptibility captures the relevance of the health issue to the general population and the likelihood that members of the general population may encounter or be affected by it.

We used GLM-4.7-FlashX to code the article headlines automatically. Based solely on the headline text, the model first identified the focal health topic and then independently rated topic health severity and topic health susceptibility on separate 1–5 scales. Higher scores indicated more serious potential health consequences and broader population susceptibility, respectively. The prompt instructed the model to disregard exaggeration, fear appeals, urgency cues, and other clickbait language and to evaluate the underlying health issue rather than the headline’s presentation style. Topic health risk was calculated as the equally weighted mean of the severity and susceptibility scores. Because both component scores range from 1 to 5, THR also ranges from 1 to 5, with higher values indicating greater topic health risk.

To assess the reliability and validity of the topic health risk (THR) measure, we conducted both human validation and cross-model validation. We randomly selected 600 article headlines from the analytical sample and asked two independent human coders, who had been trained using the same coding protocol, to evaluate topic severity and susceptibility using the same 1–5 scoring criteria applied in the LLM-based measurement. The coders subsequently worked independently and had no access to the LLM-generated scores or each other’s ratings. We further applied the alternative LLM (DeepSeek V4.1 Flash) to all 34,341 eligible article headlines. As shown in Table 1, agreement between the two human coders was high (weighted κ=0.804, $r_{s}=0.782$, mean absolute error (MAE) =0.463). The original GLM-based THR scores also showed strong agreement with the human ratings, with weighted κ ranging from 0.895 to 0.899, Spearman correlations from 0.879 to 0.891, and MAE from 0.311 to 0.316 across the two coders. The somewhat higher LLM–human agreement relative to human–human agreement may reflect differences between the two coders in their judgments of some borderline headlines, while the model remained closely aligned with each coder overall. An alternative LLM produced similarly strong agreement with human ratings (weighted κ=0.832–0.847, $r_{s}=0.810$–0.832, MAE =0.408–0.418). Cross-model agreement was also high (weighted κ=0.882, $r_{s}=0.850$, MAE =0.235).

### 4.3. Control Variables

To minimize potential confounding from content presentation characteristics, we included control variables capturing three dimensions: visual presentation, headline language, and source cues.

#### 4.3.1. Image Attractiveness

We measured image attractiveness using the Neural Image Assessment (NIMA) model (Talebi & Milanfar, 2018). NIMA was trained on the Aesthetic Visual Analysis (AVA) dataset, which contains large-scale human aesthetic evaluations (Murray et al., 2012). For each image, the model predicts a probability distribution over aesthetic ratings ranging from 1 to 10.

For each cover image, we calculated its aesthetic score as the expected value of NIMA’s predicted 1–10 rating distribution. Article-level image attractiveness was then obtained by averaging the aesthetic scores of the three cover images. Higher values indicate greater average aesthetic attractiveness across the three cover images.

#### 4.3.2. Headline Clickbait Intensity

We define headline clickbait intensity as the extent to which a headline uses exaggeration, suspense, information omission, emotional stimulation, numerical promises, and related linguistic strategies to attract clicks. The construct was measured using a five-level ordinal scale. The training data comprised approximately 2000 human-labeled headlines and 10,000 headlines labeled by a large language model, which served as gold-standard and silver-standard labels, respectively.

We trained an ordinal MacBERT classification model to estimate headline clickbait intensity (W. Cao et al., 2020; Cui et al., 2020). The model was first trained on the silver-standard data to learn clickbait-related linguistic patterns and was then fine-tuned exclusively on the human-labeled training set. Early stopping and probability calibration were performed based on performance on the human-labeled validation set.

The calibrated probabilities for the five ordinal categories were converted into a continuous headline clickbait intensity score using their probability-weighted expected value. The resulting HCI score ranges from 1 to 5, with higher values indicating greater clickbait intensity.

Model performance was evaluated using an independent human-labeled test set. Compared with a MacBERT baseline trained exclusively on the human-labeled gold-standard data, pretraining with the large language model-generated silver-standard data increased quadratic-weighted Cohen’s κ from 0.767 to 0.824 and Spearman’s correlation coefficient from 0.790 to 0.850. It also reduced the mean absolute error for the continuous score by approximately 14.5%. These results indicate that silver-standard pretraining provided useful incremental information, whereas subsequent fine-tuning on the gold-standard data helped correct potential systematic errors in the silver-standard labels, improving the accuracy and stability of the clickbait-intensity measure.

#### 4.3.3. Headline Emotional Arousal

Headline emotional arousal captures the degree to which the language of a headline evokes emotional activation, such as excitement, tension, or alertness. Arousal is an important dimension of emotional experience (Russell, 1980). To account for the potential influence of emotional activation on attention and clicking behavior, we measured headline emotional arousal using a lexicon-based approach.

Specifically, we used the simplified Chinese affective word norms developed by Xu et al. (2022). The database contains 11,310 simplified Chinese words evaluated by native Chinese speakers and provides an arousal rating ranging from 0 to 4 for each word. A score of 0 represents a very low level of emotional activation, whereas a score of 4 represents a very high level.

Before lexicon matching, we applied Unicode normalization to all headlines to reduce matching errors caused by differences in character encoding and formatting. Headline emotional arousal was then calculated as the mean arousal rating of all headline words matched to the lexicon. Higher HEA values indicate greater overall emotional activation conveyed by the headline.

#### 4.3.4. Headline Readability

Headline readability captures the amount of cognitive effort required for a general reader to understand a headline. Because news headlines are typically short, we followed the feature-based approach used in Chinese readability research (W. Li et al., 2022) and constructed a composite measure based on lexical difficulty, medical-term density, and headline length.

First, we measured lexical difficulty using the SUBTLEX-CH Chinese word-frequency database (Cai & Brysbaert, 2010). Suppose that headline *i* contains $n_{i}$ valid words and that $f_{ij}$ denotes the frequency per million words of the *j*th word. The mean lexical difficulty of the headline is defined as

(3) $${\mathrm{LexDiff}}_{i}=\frac{1}{n_{i}}\sum_{j=1}^{n_{i}}-\ln\left(\frac{\max(f_{ij},0.01)}{10^{6}}\right).$$

Second, we constructed a domain-specific health and medical terminology lexicon based on the THUOCL medical lexicon (Han et al., 2016). We supplemented the original lexicon with terms related to traditional Chinese medicine, health maintenance, nutritional indicators, and common chronic diseases. After data cleaning and deduplication, the resulting lexicon contained 18,865 terms. Medical-term density, denoted by ${\mathrm{MedRatio}}_{i}$, was defined as the proportion of valid headline words matched to the domain-specific medical lexicon. Third, headline length, denoted by ${\mathrm{Length}}_{i}$, is measured as the number of characters remaining after spaces and punctuation marks are removed.

To place the three components on a common scale, we standardized them using Z-scores and combined them with equal weights. The resulting reading-difficulty index was multiplied by −1 so that higher values indicate greater readability:

(4) $${\mathrm{HR}}_{i}=-\frac{1}{3}[Z({\mathrm{LexDiff}}_{i})+Z({\mathrm{MedRatio}}_{i})+Z({\mathrm{Length}}_{i})].$$

Higher values of ${\mathrm{HR}}_{i}$ indicate greater headline readability for a general reader.

#### 4.3.5. Health-Related Source Cue

In online health information environments, information sources and their associated expertise cues can influence users’ assessments of information credibility and quality. Prior research suggests that users consider source expertise, trustworthiness, and authority when evaluating online health information and tend to place greater trust in professional sources such as physicians and healthcare institutions (Dutta-Bergman, 2003; Sbaffi & Rowley, 2017; Y. Sun et al., 2019).

To account for the potential influence of health-related expertise cues conveyed by author names, we included a health-related source cue variable. Because users can observe an author’s displayed name before clicking on an article, this measure captures whether the displayed name itself communicates an identifiable health- or medical-related cue rather than whether the author actually possesses a particular professional identity.

We used GLM-4.7-FlashX to classify the displayed author names. The model was instructed to rely exclusively on the name text and determine whether it contained an identifiable health- or medical-related cue. Such cues included references to healthcare professionals, such as physicians, nurses, or nutritionists; healthcare institutions or clinical departments; diseases or medical topics; and health education or wellness information. When no health-related attribute could be clearly inferred from the displayed name, the author name was conservatively classified as not health-related. The resulting health-related source cue variable was coded as 1 when an identifiable health- or medical-related cue was present in the displayed author name and 0 otherwise.

Table 2 summarizes the definitions and measurement procedures for all variables included in the empirical analysis.

### 4.4. Empirical Analysis

#### 4.4.1. Model Specification

Using the number of clicks recorded on Day 1 after publication as the number of successes and the corresponding number of impressions as the number of trials, we estimated a beta-binomial model of the probability that an impression results in a click. The model preserves the binomial structure under which the number of clicks cannot exceed the number of impressions, while incorporating a dispersion parameter, ρ, to accommodate between-article heterogeneity beyond that allowed by the standard binomial distribution (Williams, 1982). All models include author fixed effects and publication-date fixed effects, with standard errors clustered at the author level.

Let ${\mathrm{Click}}_{i}$ and ${\mathrm{Imp}}_{i}$ denote the numbers of clicks and impressions for article *i*, respectively. Let $X_{i}$ denote image–text congruence, $C_{i}$ denote inter-image coherence, and $R_{i}$ denote topic health risk. All continuous predictors were standardized using Z-scores before the quadratic and interaction terms were constructed. The full model is specified as follows:

(5) $$\begin{array}{cc} {\mathrm{Click}}_{i}\; & ∼\mathrm{BetaBinomial}\left({\mathrm{Imp}}_{i},p_{i},\rho \right),\\ \mathrm{logit}(p_{i})\; & =\alpha_{a(i)}+\delta_{t(i)}+\gamma^{T}{\mathrm{Controls}}_{i}+\beta_{1}X_{i}+\beta_{2}C_{i}+\beta_{3}C_{i}^{2}+\beta_{4}R_{i}\\ \; & \;+\beta_{5}X_{i}R_{i}+\beta_{6}C_{i}R_{i}+\beta_{7}C_{i}^{2}R_{i}. \end{array}$$

Here, $p_{i}$ is the conditional probability that an impression of article *i* results in a click. The parameter ρ is the beta-binomial overdispersion parameter. Under this parameterization, the conditional variance of the article-level click count is $\mathrm{Var}\left({\mathrm{Click}}_{i}|{\mathrm{Imp}}_{i},p_{i},\rho \right)={\mathrm{Imp}}_{i}p_{i}\left(1-p_{i}\right)\left[1+\left({\mathrm{Imp}}_{i}-1\right)\rho \right]$, where ${\mathrm{Imp}}_{i}p_{i}\left(1-p_{i}\right)$ is the corresponding binomial variance. The terms $\alpha_{a(i)}$ and $\delta_{t(i)}$ represent author and publication-date fixed effects, respectively. The control vector ${\mathrm{Controls}}_{i}$ includes image attractiveness, headline clickbait intensity, headline emotional arousal, headline readability, and the health-related source cue.

#### 4.4.2. Empirical Results

The beta-binomial regression results are reported in Table 3. Model fit improved consistently as the core explanatory variables and interaction terms were added. The McFadden-type pseudo-$R^{2}$ increased from 0.0005 in M1 to 0.0110 in M5, while the AIC decreased from 430,593.7 to 426,143.1 and the BIC decreased from 449,533.8 to 445,133.9.

**Verification-Oriented Congruence and CTR.** M1 shows that, after controlling for article characteristics, author fixed effects, and publication-date fixed effects, the coefficient of image–text congruence is positive and statistically significant (β=0.059, p<0.001). The coefficient remains positive and significant as inter-image coherence, topic health risk, and the corresponding interaction terms are progressively added to the model. In the full specification, M5, the coefficient is 0.150 (p<0.001). Thus, holding the other variables constant, greater verification-oriented congruence between an article’s headline and its cover images is associated with a higher probability of clicking conditional on an impression.
**Nonlinear Association between Configurational Coherence and CTR.** In M2, the linear term for inter-image coherence is positive and statistically significant (β=0.083, p<0.001), whereas its squared term is negative and statistically significant (β=−0.093, p<0.001). In the full specification, M5, the linear and squared terms remain significant, with coefficients of 0.061 (p<0.001) and −0.067 (p<0.001), respectively.

Following the procedure for evaluating nonlinear relationships proposed by Haans et al. (2016), we further examined the turning point and the slopes at both ends of the observed range. At the mean level of topic health risk, the turning point estimated from M5 is 0.454 standard deviations, with a 95% confidence interval of [0.389,0.520]. This turning point lies within the observed range of standardized inter-image coherence, [−3.403,3.376]. At the 5th percentile of inter-image coherence, the slope on the log-odds scale is 0.283, with a 95% confidence interval of [0.239,0.326], indicating a significantly positive slope. At the 95th percentile, the slope is −0.169, with a 95% confidence interval of [−0.201,−0.138], indicating a significantly negative slope. The endpoint slopes calculated at the observed minimum and maximum are 0.520 and −0.394, respectively, and both are statistically significant at p<0.001. These results confirm an inverted U-shaped association between configurational coherence and CTR over the observed range.

**Moderating Role of Topic Health Risk in the Association between Verification-Oriented Congruence and CTR.** In M4, the interaction between image–text congruence and topic health risk is positive and statistically significant (β=0.178, p<0.001). The interaction remains positive and significant in M5 after all interaction terms are included (β=0.143, p<0.001).

The simple-slope analysis shows that, at a low level of topic health risk (−1 SD), the slope of verification-oriented congruence is 0.008, with a 95% confidence interval of [−0.007,0.022], and is therefore not statistically distinguishable from zero. At the mean level of topic health risk, the slope increases to 0.150, with a 95% confidence interval of [0.137,0.163] (p<0.001). At a high level of topic health risk (+1 SD), the slope further increases to 0.293, with a 95% confidence interval of [0.271,0.314] (p<0.001).

**Figure 2:**
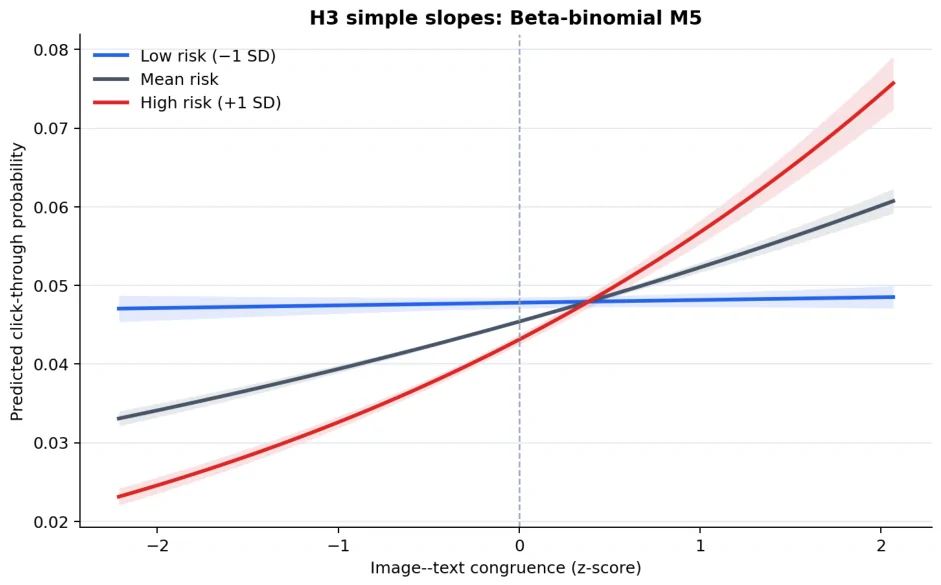
Predicted CTR by image–text congruence and topic health risk.

As shown in Figure 2, the predicted CTR curve is nearly flat under the low-risk condition but rises markedly with verification-oriented congruence under the high-risk condition. These results indicate that the positive association between verification-oriented congruence and CTR becomes stronger as topic health risk increases.

**Moderating Role of Topic Health Risk in the Association between Configurational Coherence and CTR.** In M5, the coefficient of the interaction between inter-image coherence and topic health risk is −0.014 (p<0.001), whereas the coefficient of the interaction between squared inter-image coherence and topic health risk is 0.044 (p<0.001). The negative coefficient of the squared inter-image coherence term, combined with its positive interaction with topic health risk, indicates that the absolute magnitude of the negative curvature decreases as topic health risk increases.

**Figure 3:**
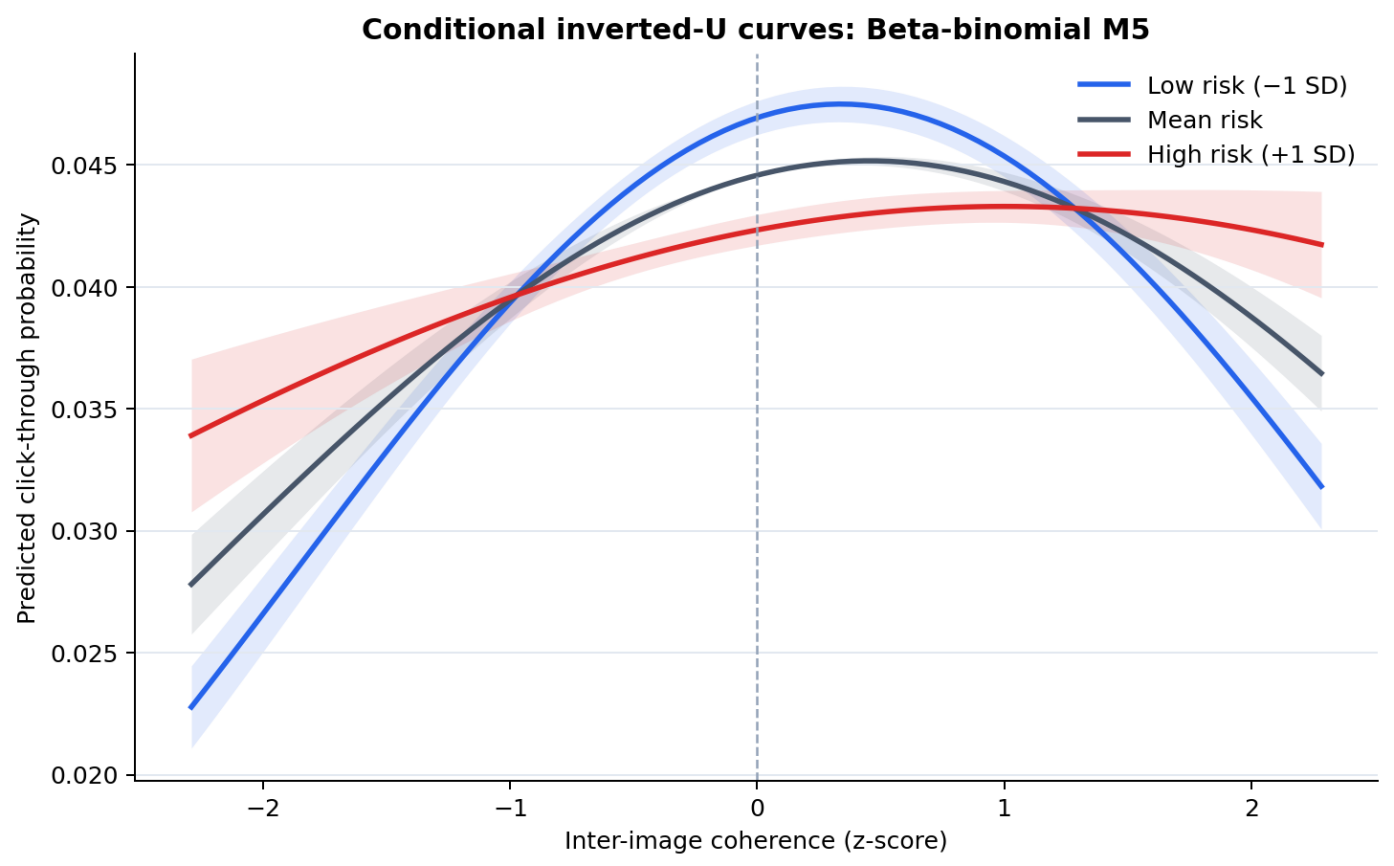
Predicted CTR by inter-image coherence and topic health risk.

The conditional-curve analysis shows that the quadratic coefficients of configurational coherence are −0.111, −0.067, and −0.024 under the low-risk (−1 SD), mean-risk, and high-risk (+1 SD) conditions, respectively. As shown in Figure 3, the declining right-hand side of the inverted U-shaped curve becomes progressively flatter as topic health risk increases.

At the 95th percentile of inter-image coherence, the slope on the log-odds scale is −0.305 under the low-risk condition, with a 95% confidence interval of [−0.344,−0.267]. At the mean level of topic health risk, the corresponding slope is −0.169, with a 95% confidence interval of [−0.201,−0.138]. Under the high-risk condition, the slope is −0.034, with a 95% confidence interval of [−0.076,0.009], and is therefore not statistically distinguishable from zero. The difference between the right-hand slopes under the high- and low-risk conditions is 0.272, with a 95% confidence interval of [0.220,0.323] (p<0.001). These results indicate that the decline in CTR associated with high configurational coherence is weaker when topic health risk is higher.

Because THR is measured using an LLM, we conducted an additional robustness check to assess whether the moderation results depend on the specific language model used to construct this variable. We remeasured THR for the full sample using an alternative LLM and re-estimated the complete M5 specification while keeping the sample, control variables, fixed effects, and model specification unchanged. The ITC × THR interaction remained positive and statistically significant (β=0.1302, p<0.001). The IIC × THR interaction remained negative (β=−0.0146, p<0.001), while the IIC^2^× THR interaction remained positive (β=0.0455, p<0.001). These estimates are closely aligned in both direction and magnitude with the original M5 coefficients, providing further evidence that the findings for H3 and H4 are not driven by the use of a particular LLM to measure topic health risk.

#### 4.4.3. Practical Magnitude of the Estimated Effects

To assess the practical magnitude of the associations in M5, we translated the beta-binomial estimates into average predicted click-through rates (CTRs). Table 4 shows that, with observed THR values retained, increasing image–text congruence (ITC) by one standard deviation raises average predicted CTR from 4.1835% to 4.9062%, corresponding to an increase of 0.7227 percentage points (95% CI [0.2714, 1.1740]). This increase is equivalent to 17.46% of the sample mean CTR of 4.1393%. The magnitude increases with topic health risk: the corresponding changes are 0.0314 percentage points at low THR (−1 SD), 0.6513 percentage points at mean THR, and 1.3605 percentage points at high THR (+1 SD).

Table 5 reports probability-scale differences for inter-image coherence (IIC), using IIC =0 SD as the reference within each THR scenario. At mean THR, setting IIC to −1 SD lowers predicted CTR from 4.5589% to 4.0360%, a decrease of 0.5229 percentage points (95% CI [−0.8697, −0.1760]). Setting IIC to +1 SD yields a predicted CTR of 4.5323%, only 0.0266 percentage points below the reference. The high-IIC contrast becomes progressively weaker as THR increases: the difference at IIC =+1 SD is −0.1603 percentage points under low THR, −0.0266 at mean THR, and 0.1003 under high THR. This pattern is consistent with the conditional turning-point analysis, which indicates that higher THR shifts the peak of the IIC–CTR relationship toward higher coherence and weakens the decline at high IIC.

#### 4.4.4. Robustness Checks

To assess whether the findings depend on a particular model specification, measurement approach, treatment of extreme observations, or outcome observation window, we conducted two sets of robustness checks: alternative model specifications and alternative research-design choices. The core findings remain consistent across these analyses.

First, robustness to alternative model specifications. We re-estimated the full model using four alternative specifications. Specifically, we employed: (1) a beta-binomial model with author random intercepts and publication-date fixed effects, using the full sample, including authors who published only one article, to examine whether the findings remain stable when author heterogeneity is modeled as a random effect; (2) a Poisson pseudo-maximum likelihood (PPML) model with $\log({\mathrm{Imp}}_{i})$ as an offset, along with author and publication-date fixed effects, treating the number of clicks as a count outcome and the number of impressions as the scale of exposure to click opportunities; (3) a standard binomial logit model in which clicks are treated as successes and impressions as trials, with author and publication-date fixed effects and authors appearing only once excluded; and (4) a negative binomial regression model with $\log({\mathrm{Imp}}_{i})$ as an offset and author and publication-date fixed effects to further accommodate potential overdispersion in the click-count outcome. These models modify the treatment of author heterogeneity, the assumed distribution of the outcome, and the way impressions are incorporated into the analysis. The results are reported in Table 6. Comparing the directions and statistical significance of the focal coefficients and interaction terms across these specifications allows us to determine whether the findings depend excessively on the beta-binomial distribution or the use of author fixed effects.

Second, robustness to alternative research-design choices. We conducted four additional tests addressing variable measurement, extreme observations, and outcome observation windows. Specifically, we: (1) replaced the original image–text congruence and inter-image coherence measures with alternative measures calculated using AltCLIP and DINOv2, respectively, to examine whether the findings depend on a particular representation model; the detailed measurement procedures are provided in Appendix A; (2) excluded articles in the top 1% of the impression distribution and continued to exclude authors appearing only once, thereby reducing the influence of a small number of articles with exceptionally high exposure; (3) replaced the Day 1 outcomes with cumulative clicks and impressions measured on Day 7 to evaluate whether the findings hold during the short-term accumulation phase; and (4) used cumulative clicks and impressions measured on Day 21 to determine whether the findings persist after article traffic has largely entered the plateau phase. All four tests were conducted using the same beta-binomial framework as the main analysis, while retaining the control variables, author fixed effects, and publication-date fixed effects wherever possible. The results are reported in Table 7. Together, these tests reduce concerns that the findings are driven by a specific measurement method, exceptionally high-exposure articles, or the timing of outcome measurement.

#### 4.4.5. Sensitivity Analyses

To assess whether the main findings are sensitive to differences in algorithmically allocated exposure, we conducted two additional sensitivity analyses. First, we augmented the original beta-binomial M5 specification with Day-1 impression-decile indicators while retaining the same analytical sample, control variables, author fixed effects, publication-date fixed effects, and author-clustered standard errors. This specification more flexibly accounts for differences in exposure intensity across articles. Second, we divided the sample into low (≤2802 impressions), middle (2803–7957 impressions), and high (≥7958 impressions) exposure groups and re-estimated M5 within each group after excluding authors represented by only one article in the corresponding subsample.

As shown in Table 8, the main results remain stable after accounting for exposure intensity. In the exposure-adjusted model, image–text congruence remains positively associated with CTR (β=0.117, p<0.001), while inter-image coherence retains the inverted U-shaped pattern ($\beta_{\mathrm{IIC}}=0.048$, p<0.001; $\beta_{{\mathrm{IIC}}^{2}}=-0.057$, p<0.001). The moderation results also remain consistent: the ITC × THR interaction is positive (β=0.116, p<0.001), the IIC × THR interaction is negative (β=−0.016, p<0.001), and the IIC^2^ × THR interaction is positive (β=0.039, p<0.001). These estimates closely reproduce the direction of the original M5 results after exposure intensity is explicitly controlled.

The exposure-stratified analyses yield a similar pattern. ITC remains positive and statistically significant in the low-, middle-, and high-exposure groups (β=0.171, 0.101, and 0.083, respectively; all p<0.001), and the positive linear and negative quadratic terms of IIC remain significant across all three groups, supporting the inverted U-shaped relationship. The ITC × THR interaction is likewise positive and significant across low, middle, and high exposure (β=0.190, 0.096, and 0.102, respectively; all p<0.001). The moderation of the IIC relationship is also consistent across exposure levels. The IIC × THR interaction remains negative in the low-, middle-, and high-exposure groups (β=−0.009, −0.012, and −0.026, respectively), while the IIC^2^ × THR interaction remains positive and significant (β=0.017, 0.039, and 0.049, respectively). Thus, across different levels of exposure, higher THR is consistently associated with a weaker negative curvature of the IIC–CTR relationship. Taken together, these sensitivity analyses suggest that the main findings are robust to alternative ways of accounting for observable differences in exposure intensity.

## 5. Discussion

### 5.1. Summary of Findings

This study examined the associations between two forms of cover consistency and article click-through rate (CTR) on a digital health platform, as well as the moderating role of topic health risk. The results show that verification-oriented congruence between an article’s headline and its cover images is positively associated with CTR. By contrast, configurational coherence among multiple cover images has an inverted U-shaped association with CTR: moderate configurational coherence is associated with a higher CTR, whereas both low and high coherence are associated with lower CTRs.

Topic health risk further moderates these relationships. As topic health risk increases, the positive association between verification-oriented congruence and CTR becomes stronger. At the same time, the decline in CTR associated with high configurational coherence is weaker for high-risk topics. Taken together, cover consistency is neither a unitary content attribute nor universally beneficial. Its association with CTR depends on whether consistency occurs between the headline and images or within the set of cover images, as well as on the health risk associated with the topic.

### 5.2. Theoretical Contributions

This study makes three primary theoretical contributions. First, this study extends research on digital health information to the stage of pre-click information selection. Prior research has primarily examined how information presentation and visual content relate to user understanding and engagement (Z. Jiang & Benbasat, 2007; Y. Li & Xie, 2020). We conceptualize a multi-image cover as a compressed interface-level representation of article content and examine how the organization of its headline and images is associated with users’ initial click-through behavior. Moreover, by modeling clicks relative to article impressions, this study examines engagement conditional on observed visibility without identifying the platform’s allocation process. It thereby provides a more differentiated understanding of user engagement on digital health platforms.

Second, this study refines the construct of multimodal consistency. In contrast to research that focuses primarily on individual image characteristics or general image–text congruence (J. Cao et al., 2025; Y. Li & Xie, 2020), we distinguish image–text congruence, which serves a topic-verification function, from inter-image coherence, which concerns the organization of the visual set. The two forms of consistency exhibit different associations with CTR: the former has a positive association, whereas the latter has an inverted U-shaped association. These distinct patterns suggest that cross-modal semantic alignment and within-modal visual organization may reflect different information-processing benefits and costs. Alignment between a headline and its images can clarify the article’s topic, whereas similarity among images can provide a common theme but also limit the distinct information conveyed by each image. Multimodal consistency should therefore not be treated as a single, undifferentiated content attribute but should instead be distinguished according to its relational structure and informational function.

Third, this study identifies a nonlinear association and a health-risk boundary condition. Research on processing fluency and cognitive fit suggests that coordinated information representations can reduce the cognitive effort required to process information (Reber et al., 2004; Vessey, 1991). We find an inverted U-shaped association between inter-image coherence and CTR, with the estimated turning point and endpoint slopes satisfying established criteria for evaluating nonlinear relationships (Haans et al., 2016). This finding points to a potential trade-off between visual coherence and informational richness. Furthermore, as topic health risk increases, the positive association between image–text congruence and CTR becomes stronger, while the decline on the right-hand side of the inter-image coherence curve becomes weaker. These findings extend research on risk information processing (Griffin et al., 1999) to the context of multimodal information design and indicate that the association between cover consistency and CTR is contingent on the health-risk context. The implication is not that more consistency is always preferable under higher risk; rather, risk changes the relative importance of headline–image alignment and the balance between coherence and informational differentiation within an image set.

These distinctions generate testable directions for further research. A factorial experiment could vary headline–image congruence and inter-image coherence independently for the same health topics while holding exposure position constant. If congruence works through topic recognition, its effects should be accompanied by faster or more accurate identification of an article’s subject. If high inter-image coherence reduces the value of additional images, it should also reduce perceived informational diversity. Collecting evaluations of the same previews from multiple readers would reveal whether these responses differ by health concerns, health literacy, or other user characteristics. Such studies could compare initial clicks with attention, comprehension, and trust, thereby testing the proposed mechanisms rather than inferring them from CTR alone.

### 5.3. Practical Implications

#### 5.3.1. Implications for Content Creators

Content creators should consider image–text congruence and inter-image coherence as separate design objectives. First, cover images should accurately represent the focal object, health issue, or primary information presented in the article. Creators should avoid using images that are visually attractive but only weakly related to the article’s content. Semantic alignment between the headline and cover images is particularly important for high-risk health topics. For content concerning disease warnings, serious symptoms, treatment options, or medication safety, cover images should enable users to identify the focal health risk quickly rather than relying on ambiguous, sensational, or potentially misleading visual cues.

Second, multiple cover images should share a common theme while serving distinct informational functions. For low-risk, everyday wellness content, creators can increase informational richness by presenting different settings, objects, or perspectives and avoiding substantial repetition across the three images. For high-risk health content, moderate repetition of key symptoms, affected body areas, or protective behaviors may serve a confirmatory function. Consequently, high inter-image coherence may not produce the same reduction in engagement for high-risk topics as it does for low-risk topics. This does not mean, however, that greater repetition is always preferable in high-risk contexts. A more appropriate design would keep a consistent focal theme while making a distinct contribution with each image. Such design guidance should be evaluated against accurate health communication, not clicks alone.

#### 5.3.2. Implications for Digital Platforms

Digital platforms could incorporate image–text congruence and inter-image coherence into content-quality assessment and creator-support systems. For example, multimodal models could identify cover images that diverge semantically from an article’s headline or focal topic and prompt creators to revise them. Inter-image coherence, however, should not be evaluated using a simple "higher is better" rule. Platform systems should instead identify both insufficient coherence and excessive visual repetition. Topic health risk could inform how platforms evaluate the balance between coherence and informational differentiation. For high-risk content, platforms should prioritize semantic accuracy between headlines and images while allowing moderate visual repetition that serves a confirmatory function. For low-risk content, greater informational differentiation across images may help preserve novelty and user attention.

Before adopting these criteria as platform rules, providers could test alternative cover designs in controlled experiments. Evaluation should include whether previews accurately represent the underlying article and whether users understand and trust the information after clicking. This would help platforms distinguish designs that merely attract an initial click from those that also support responsible health communication.

### 5.4. Limitations and Future Research

This study has three primary limitations. First, it relies on observational platform data. Although the models account for author fixed effects, publication-date fixed effects, and multiple content characteristics, and although the findings are evaluated through a series of robustness checks, endogeneity arising from unobserved content characteristics and the platform’s recommendation mechanisms cannot be completely ruled out. The relatively modest McFadden-type pseudo-$R^{2}$ values also suggest that substantial variation in article-level CTR remains unexplained. Future research could conduct randomized cover-image experiments or platform-based A/B tests, or exploit exogenous changes in recommendation rules to construct quasi-natural experiments.

Second, the sample was drawn from a single Chinese digital platform and was limited to articles using multi-image covers. Platforms differ in interface design, recommendation logic, user populations, and content-consumption goals. Future studies could compare news and information platforms, social media platforms, and online medical communities and extend the analysis to infographics, short-form videos, and other forms of multimodal content.

Third, this study uses computational methods to measure variables and uses article-level click-through rate (CTR) as the behavioral indicator of initial user engagement. These measures enable large-scale analysis but cannot fully capture users’ perceived risk, attentional processes, post-click reading, immediate exits, content comprehension, trust, or subsequent health behavior. They also cannot capture heterogeneity in how different users or user segments perceive the same content. Future research could collect actual user evaluations in smaller samples and combine surveys, experiments, eye-tracking, and user-level behavioral data to examine heterogeneity across user segments and distinguish topic-level health risk from subjective risk perception. It could also examine the longer-term effects of multimodal information design on reading duration, knowledge acquisition, information trust, sharing behavior, and health-related decision making.

## 6. Conclusions

This study examined how the relational organization of headline and cover-image cues is associated with users’ click-through behavior in online health communication. Using large-scale behavioral data from 34,341 health-related articles, we distinguished image–text congruence from inter-image coherence and found that these two forms of multimodal consistency exhibit different associations with CTR. Image–text congruence is positively associated with CTR, whereas inter-image coherence shows an inverted U-shaped association. Topic health risk further conditions these relationships: as topic health risk increases, the positive association between image–text congruence and CTR becomes stronger, while the decline in CTR at high levels of inter-image coherence becomes weaker.

These findings suggest that multimodal consistency should not be treated as a single "more is better" design principle. Cross-modal alignment and within-image-set coherence represent distinct dimensions of multimodal design, and their associations with user selection vary across health-risk contexts. For content creators and digital platforms, this implies that headline–image alignment and inter-image coherence should be considered separately rather than optimized according to a uniform consistency rule. By focusing on the pre-click stage, this study contributes to understanding how users respond to compressed multimodal previews before accessing full health content. At the same time, CTR captures only an initial article-opening decision and does not indicate whether users subsequently read, comprehend, or remain engaged with the article content. Future research should therefore examine whether the same design features also influence attention, reading duration, comprehension, trust, and downstream health-related behavior.

## Figures and Tables

**Table 1:** Agreement in THR scores.

Comparison	*N*	Weighted κ	Spearman $r_{s}$	MAE
Human coder agreement	600	0.804	0.782	0.463
GLM vs. Human	600	0.895–0.899	0.879–0.891	0.311–0.316
Alternative LLM vs. Human	600	0.832–0.847	0.810–0.832	0.408–0.418
GLM vs. Alternative LLM	34,341	0.882	0.850	0.235

**Table 2:** Definitions and measurements of the main variables.

Variable	Definition	Measurement
**Outcome and Exposure Variables**		
Click Count (Click)	The cumulative number of article-opening clicks recorded within a specified observation window.	The Day 1 click count is used in the main analysis; the Day 7 and Day 21 click counts are used in the robustness analyses.
Impression Count (Imp)	The cumulative number of times an article was displayed to platform users within the corresponding observation window.	Used as the number of trials in the beta-binomial model and as the exposure term in the count-data models.
**Independent Variables**		
Image–Text Congruence (ITC)	The degree of cross-modal semantic alignment between an article’s headline and its three cover images.	Mean cosine similarity between the headline embedding and the embeddings of the three cover images, estimated using Chinese-CLIP.
Inter-Image Coherence (IIC)	The degree of internal semantic coherence among the three cover images associated with the same article.	Mean pairwise cosine similarity among the embeddings of the three cover images, estimated using Chinese-CLIP.
**Moderating Variable**		
Topic Health Risk (THR)	The overall level of health risk associated with the health topic represented by an article’s headline.	Equal-weight mean of the topic severity and topic susceptibility ratings.
**Control Variables**		
Image Attractiveness (IA)	The overall aesthetic appeal of the three images accompanying an article.	Mean aesthetic score predicted by the Neural Image Assessment (NIMA) model across the three cover images.
Headline Clickbait Intensity (HCI)	The extent to which a headline exhibits linguistic characteristics intended to attract clicks.	Continuous expected score generated by an ordinal MacBERT model trained on human- and LLM-labeled data.
Headline Emotional Arousal (HEA)	The degree of emotional activation conveyed by an article’s headline.	Mean arousal score of the matched headline words, calculated using a Chinese valence–arousal lexicon.
Headline Readability (HR)	The ease with which an article’s headline can be read and understood.	Standardized composite index based on word frequency, medical-term density, and headline length.
Health-Related Source Cue (HSC)	Whether the displayed author name provides an observable health- or medical-related source cue.	Binary indicator equal to 1 when the author name contains health-, medical-, nutrition-, or wellness-related semantic cues, and 0 otherwise.

**Table 3:** Beta-binomial regression results.

Variable	M1	M2	M3	M4	M5
Image–text congruence	0.059 ***	0.131 ***	0.159 ***	0.158 ***	0.150 ***
	(0.004)	(0.007)	(0.007)	(0.007)	(0.007)
Inter-image coherence		0.083 ***	0.079 ***	0.063 ***	0.061 ***
		(0.006)	(0.006)	(0.006)	(0.006)
Inter-image coherence squared		−0.093 ***	−0.080 ***	−0.073 ***	−0.067 ***
		(0.006)	(0.005)	(0.006)	(0.006)
Topic health risk (THR)			0.089 ***	−0.001	−0.055 ***
			(0.006)	(0.007)	(0.008)
Image–text congruence × THR				0.178 ***	0.143 ***
				(0.005)	(0.006)
Inter-image coherence × THR					−0.014 ***
					(0.004)
Inter-image coherence squared × THR					0.044 ***
					(0.004)
Control variables	Yes	Yes	Yes	Yes	Yes
Author fixed effects	Yes	Yes	Yes	Yes	Yes
Publication-date fixed effects	Yes	Yes	Yes	Yes	Yes
SEs clustered by author	Yes	Yes	Yes	Yes	Yes
Articles	34,341	34,341	34,341	34,341	34,341
Authors	2184	2184	2184	2184	2184
McFadden-type pseudo-$R^{2}$	0.0005	0.0029	0.0039	0.0104	0.0110
Log likelihood	−213,053.8	−212,554.8	−212,327.0	−210,936.2	−210,822.5
AIC	430,593.7	429,599.6	429,146.0	426,366.4	426,143.1
BIC	449,533.8	448,556.6	448,111.4	445,340.3	445,133.9
Overdispersion parameter, ρ	0.0230	0.0223	0.0220	0.0202	0.0200

*Notes:* *** p<0.001.

**Table 4:** Average predicted CTR changes in ITC.

THR Scenario	ITC Change	Baseline CTR	New CTR	Change (pp)	95% CI (pp)
Observed THR	+1 SD	4.1835%	4.9062%	0.7227	[0.2714, 1.1740]
Low THR (−1 SD)	+1 SD	4.3469%	4.3784%	0.0314	[−0.0314, 0.0942]
Mean THR (0 SD)	+1 SD	4.2761%	4.9274%	0.6513	[0.2296, 1.0730]
High THR (+1 SD)	+1 SD	4.3109%	5.6714%	1.3605	[0.4945, 2.2266]

**Table 5:** Average predicted CTR differences across IIC scenarios.

THR Scenario	IIC Level	Baseline CTR	New CTR	Change (pp)	95% CI (pp)
Low THR (−1 SD)	−1 SD	4.7792%	4.0072%	−0.7720	[−1.2760, −0.2680]
Low THR (−1 SD)	+1 SD	4.7792%	4.6189%	−0.1603	[−0.2790, −0.0417]
Mean THR (0 SD)	−1 SD	4.5589%	4.0360%	−0.5229	[−0.8697, −0.1760]
Mean THR (0 SD)	+1 SD	4.5589%	4.5323%	−0.0266	[−0.0695, 0.0164]
High THR (+1 SD)	−1 SD	4.4258%	4.1381%	−0.2877	[−0.5009, −0.0745]
High THR (+1 SD)	+1 SD	4.4258%	4.5261%	0.1003	[0.0194, 0.1812]

**Table 6:** Robustness checks using alternative model specifications.

Variable	(1)	(2)	(3)	(4)
	Beta-Binomial RE	PPML FE	Binomial Logit FE	NB2 FE
Image–text congruence	0.142 ***	0.116 ***	0.124 ***	0.152 ***
	(0.005)	(0.030)	(0.032)	(0.010)
Inter-image coherence	0.054 ***	0.072 **	0.076 **	0.070 ***
	(0.005)	(0.024)	(0.025)	(0.007)
Inter-image coherence squared	−0.064 ***	−0.068 **	−0.070 **	−0.070 ***
	(0.003)	(0.023)	(0.025)	(0.006)
Topic health risk (THR)	−0.047 ***	−0.111 **	−0.118 **	−0.071 ***
	(0.005)	(0.038)	(0.041)	(0.010)
Image–text congruence × (THR)	0.137 ***	0.188 ***	0.201 ***	0.173 ***
	(0.005)	(0.025)	(0.026)	(0.009)
Inter-image coherence × (THR)	−0.011 **	−0.084 **	−0.088 **	−0.025 ***
	(0.004)	(0.029)	(0.031)	(0.005)
Inter-image coherence squared × (THR)	0.040 ***	0.058 **	0.061 **	0.048 ***
	(0.003)	(0.021)	(0.023)	(0.004)
Control variables	Yes	Yes	Yes	Yes
Author effects	Random intercept	Fixed effects	Fixed effects	Fixed effects
Publication-date fixed effects	Yes	Yes	Yes	Yes
Exposure specification	Binomial trials	$\log({\mathrm{Imp}}_{i})$ offset	Binomial trials	$\log({\mathrm{Imp}}_{i})$ offset
Standard errors	Model-based	Author-clustered	Author-clustered	Author-clustered
Articles	36,446	34,341	34,341	34,341
Authors	4289	2184	2184	2184

*Notes:* *** p<0.001; ** p<0.01.

**Table 7:** Robustness checks using alternative measurements, samples, and outcome windows.

Variable	(1)	(2)	(3)	(4)
	Alternative Measures	Top 1% Trimmed	Day 7 Outcome	Day 21 Outcome
Image–text congruence	0.204 ***	0.150 ***	0.143 ***	0.136 ***
	(0.009)	(0.007)	(0.007)	(0.006)
Inter-image coherence	0.185 ***	0.062 ***	0.057 ***	0.054 ***
	(0.011)	(0.006)	(0.006)	(0.005)
Inter-image coherence squared	−0.077 ***	−0.068 ***	−0.065 ***	−0.062 ***
	(0.005)	(0.006)	(0.005)	(0.005)
Topic health risk (THR)	0.028 **	−0.053 ***	−0.055 ***	−0.052 ***
	(0.010)	(0.008)	(0.008)	(0.007)
Image–text congruence × (THR)	0.130 ***	0.142 ***	0.139 ***	0.133 ***
	(0.006)	(0.007)	(0.006)	(0.006)
Inter-image coherence × (THR)	−0.065 ***	−0.014 ***	−0.010 **	−0.009 **
	(0.007)	(0.004)	(0.003)	(0.003)
Inter-image coherence squared × (THR)	0.037 ***	0.044 ***	0.041 ***	0.040 ***
	(0.004)	(0.004)	(0.004)	(0.004)
Measurement of focal variables	AltCLIP DINOv2	Original	Original	Original
Outcome window	Day 1	Day 1	Day 7	Day 21
Top 1% of impressions removed	No	Yes	No	No
Control variables	Yes	Yes	Yes	Yes
Author fixed effects	Yes	Yes	Yes	Yes
Publication-date fixed effects	Yes	Yes	Yes	Yes
SEs clustered by author	Yes	Yes	Yes	Yes
Articles	34,341	33,986	34,341	34,341
Authors	2184	2172	2184	2184

*Notes:* *** p<0.001; ** p<0.01.

**Table 8:** Sensitivity analyses accounting for exposure intensity.

Variable	(1)	(2)	(3)	(4)
	Exposure-Adjusted M5	Low Exposure	Middle Exposure	High Exposure
Image–text congruence	0.117 ***	0.171 ***	0.101 ***	0.083 ***
	(0.007)	(0.013)	(0.008)	(0.010)
Inter-image coherence	0.048 ***	0.038 ***	0.037 ***	0.054 ***
	(0.005)	(0.010)	(0.007)	(0.008)
Inter-image coherence squared	−0.057 ***	−0.048 ***	−0.042 ***	−0.057 ***
	(0.005)	(0.010)	(0.006)	(0.008)
Topic health risk (THR)	−0.052 ***	−0.038 *	−0.041 ***	−0.060 ***
	(0.007)	(0.016)	(0.008)	(0.009)
Image–text congruence × THR	0.116 ***	0.190 ***	0.096 ***	0.102 ***
	(0.006)	(0.016)	(0.010)	(0.009)
Inter-image coherence × THR	−0.016 ***	−0.009 *	−0.012 *	−0.026 ***
	(0.003)	(0.004)	(0.005)	(0.006)
Inter-image coherence squared × THR	0.039 ***	0.017 *	0.039 ***	0.049 ***
	(0.004)	(0.009)	(0.006)	(0.005)
Control variables	Yes	Yes	Yes	Yes
Author fixed effects	Yes	Yes	Yes	Yes
Publication-date fixed effects	Yes	Yes	Yes	Yes
SEs clustered by author	Yes	Yes	Yes	Yes
Exposure-decile indicators	Yes	No	No	No
Articles	34,341	11,090	10,921	11,048
Authors	2184	1231	898	818
Publication dates	53	53	53	53
Overdispersion parameter, ρ	0.0162	0.0120	0.0125	0.0202
Log likelihood	−207,428.1	−47,389.1	−63,144.6	−87,926.0

*Notes:* *** p<0.001; * p<0.05.

## Data Availability

The de-identified data supporting the findings of this study are available from the authors upon reasonable request. The raw data are not publicly available due to restrictions on the redistribution of platform-derived data and privacy considerations concerning potentially identifiable account information.

## Note

1 https://www.toutiao.com/.

## Appendix A. Alternative Measurements for Independent Variables

To assess whether the measurement of image–text congruence and inter-image coherence depends on the specific architecture, training data, or training objective of Chinese-CLIP, we reconstructed the two focal variables using alternative representation models. Specifically, we used AltCLIP as an alternative cross-modal encoder for image–text congruence and DINOv2 as an alternative visual encoder for inter-image coherence.

### Appendix A.1. Alternative Measure of Image–Text Congruence

For the alternative measurement of image–text congruence, we used AltCLIP to extract cross-modal semantic representations of article headlines and their corresponding cover images. AltCLIP employs XLM-R as its text encoder and uses knowledge distillation and contrastive learning to establish semantic correspondence between images and multilingual text, including Chinese text (Chen et al., 2023). AltCLIP differs from Chinese-CLIP in its text encoder, pretraining data, and training strategy. Consistent results across the two models would therefore reduce concerns that the findings are driven by the characteristics of a particular pretrained vision–language model.

Let $f_{T}^{A}\left(\cdot \right)$ and $f_{I}^{A}\left(\cdot \right)$ denote the text and image encoders of AltCLIP, respectively. After applying $L_{2}$ normalization to the model outputs, the alternative image–text congruence between the headline of article *i* and its *j*th cover image is calculated as

(A1) $$s_{ij}^{IT,A}=\frac{f_{T}^{A}{\left(T_{i}\right)}^{T}f_{I}^{A}\left(I_{ij}\right)}{{∥f_{T}^{A}\left(T_{i}\right)∥}_{2}{∥f_{I}^{A}\left(I_{ij}\right)∥}_{2}},\;j=1,2,3.$$

At the article level, the AltCLIP-based image–text congruence measure is obtained by taking the equally weighted mean of the three headline–image similarity scores:

(A2) $${\mathrm{ITC}}_{i}^{A}=\frac{1}{3}\sum_{j=1}^{3}s_{ij}^{IT,A}.$$

A higher value of ${\mathrm{ITC}}_{i}^{A}$ indicates stronger semantic alignment between the article headline and its three cover images according to AltCLIP.

### Appendix A.2. Alternative Measure of Inter-Image Coherence

For the alternative measurement of inter-image coherence, we used DINOv2 as the visual representation model. DINOv2 is pretrained through self-supervised learning on a large-scale collection of unlabeled images and can extract transferable visual features without relying on paired image–text data (Oquab et al., 2024). Using DINOv2 therefore allows us to assess whether the inter-image coherence results obtained using Chinese-CLIP depend on its image–text contrastive learning objective.

Let g(·) denote the DINOv2 image encoder. The cosine similarity between the *j*th and *k*th cover images associated with article *i* is calculated as

(A3) $$s_{ijk}^{II,D}=\frac{g{\left(I_{ij}\right)}^{T}g\left(I_{ik}\right)}{{∥g(I_{ij})∥}_{2}{∥g(I_{ik})∥}_{2}},\;j<k,\;j,k\in \left\{1,2,3\right\}.$$

Because each article contains three cover images, the DINOv2-based inter-image coherence measure is defined as the mean similarity across the three unique image pairs:

(A4) $${\mathrm{IIC}}_{i}^{D}=\frac{1}{3}\left(s_{i12}^{II,D}+s_{i13}^{II,D}+s_{i23}^{II,D}\right).$$

A higher value of ${\mathrm{IIC}}_{i}^{D}$ indicates that the three cover images convey more similar visual–semantic content according to DINOv2.
